# Soluble Receptor for Advanced Glycation End Product: A Biomarker for Acute Coronary Syndrome

**DOI:** 10.1155/2015/815942

**Published:** 2015-09-30

**Authors:** Louise J. N. Jensen, Allan Flyvbjerg, Mette Bjerre

**Affiliations:** The Medical Research Laboratory, Department of Clinical Medicine, Aarhus University, 8000 Aarhus C, Denmark

## Abstract

The receptor of advanced glycation end products (RAGE) and its ligands are linked to the pathogenesis of coronary artery disease (CAD), and circulating soluble receptor of advanced glycation end products (sRAGE), reflecting the RAGE activity, is suggested as a potential biomarker. Elevated sRAGE levels are reported in relation to acute ischemia and this review focuses on the role of sRAGE as a biomarker for the acute coronary syndrome (ACS). The current studies demonstrated that sRAGE levels are elevated in relation to ACS, however during a very narrow time period, indicating that the time of sampling needs attention. Interestingly, activation of RAGE may influence the pathogenesis and reflection in sRAGE levels in acute and stable CAD differently.

## 1. Introduction

Worldwide, cardiovascular disease (CVD) is a prominent cause of increased morbidity and mortality with a heavy burden on the health care system [1]. Even though improvements in prevention, diagnosing, and treatment of the disease have increased substantially during the last decades, more attention is needed. It is believed that inflammatory mechanisms are involved in the development of CVD [2, 3], though the detailed pathogenic role of the inflammation system is still under investigation. The receptor of advanced glycation end products (RAGE) is found to play an important role in the development of CVD [4], and the soluble RAGE (sRAGE) may to some extent reflect RAGE activity, thus increasing the value of sRAGE as a biomarker [5, 6]. This review focuses on the role of sRAGE as a biomarker for the acute coronary syndrome (ACS).

## 2. Receptor for Advanced Glycation End Products (RAGE) 

RAGE is a transmembrane receptor of the immunoglobulin superfamily composed of three domains: an extracellular domain binding to ligands, a hydrophobic membrane spanning domain, and a highly charged cytoplasmic domain essential for the intracellular signaling. RAGE is expressed in many cell types including endothelial cells, lymphocytes, monocytes, and vascular smooth muscle cells. RAGE expression is minimal under normal conditions but increases significantly during cellular stress [7, 8].

RAGE was first described as a receptor for advanced glycation end products (AGEs) and it was initially linked to hyperglycemia, diabetes, and diabetic complications [9]. However, RAGE is now characterized as a multiligand receptor [10], and, apart from AGEs, RAGE interacts with other ligands, such as the S100 proteins [11], high mobility group box 1 (HMGB1) [12, 13], and amyloids [14]. Ligand binding is described to increase RAGE activity [15, 16], which mediates proinflammatory responses [17–19] and generates oxidative stress [15, 18, 20] that may contribute to the pathogenesis of CVD. Still, the exact function in vascular pathogenesis is unclear.

Mechanistic studies showed that cardiomyocytes upregulated both RAGE and AGEs after exposure to hypoxia followed by reoxygenation. Furthermore, cardiomyocytes isolated from genetic RAGE knockout or from mice pretreated with sRAGE showed protection against cellular damage [21]. Similarly, RAGE expression increased in mice myocardium after a temporary occlusion of the left anterior descending artery compared to sham-operated animals and RAGE colocalized with apoptotic cardiomyocytes [22]. The infarct sizes diminished in RAGE knockout mice hearts exposed to I/R injury [23] and precursors of RAGE ligands were reduced [24]. Furthermore, RAGE knockout mice or mice treated with RAGE inhibitors had less impaired cardiac function [12, 23–25] and diminished atherosclerosis [18, 26]. Moreover, administration of sRAGE reduced atherosclerotic lesions in atherosclerotic and diabetic mice models [4, 27, 28]. Inhibition or deletion of RAGE suppressed proinflammatory activity and oxidative stress [12, 24, 29, 30]. Additionally, RAGE ligands are reported to be involved in monocyte migration and cholesterol efflux from macrophages, and the effect was diminished through anti-RAGE antibodies or sRAGE [31, 32].

In human settings, RAGE was highly expressed in plaques, retrieved after carotid endarterectomy, from diabetic patients compared to plaques from the nondiabetic patients [33] and RAGE was primarily associated with apoptotic smooth muscle cells and macrophages together with an increased proinflammatory response [33, 34]. Furthermore, increased RAGE mRNA was found in mononuclear cells also from patients with premature CAD when compared to cells from healthy controls [35]. Together these experimental and morphological studies point towards RAGE activation in I/R injury and atherosclerosis.

## 3. Soluble RAGE (sRAGE)

Soluble isoforms of RAGE are found in the circulation and may act as regulators of RAGE activity by competitive inhibition. These isoforms lack the intramembranous and intracellular parts of the receptor, which devoid intracellular signaling [36]. Soluble RAGE is produced in two different ways, either as a splice variant, esRAGE, from a truncated RAGE mRNA [6, 37] or as a cleaved variant. Metalloproteinases cleave sRAGE from the full-length RAGE from the cell membrane [5, 38, 39]. So far, the concentrations of soluble RAGE have been determined as esRAGE or as the total amount of sRAGE, which are positively correlated [40, 41], and esRAGE constitutes 20% of total sRAGE [41, 42]. Different functions of the secreted and the cleaved sRAGE have not yet been demonstrated. Furthermore, sRAGE may reflect enhanced activity in the RAGE system since the effects of ligand stimulation mediate sRAGE upregulation [5] and sRAGE is secreted in parallel with RAGE [5, 6]. This property makes sRAGE a valuable biomarker.

## 4. sRAGE in Patients with Acute Coronary Syndrome (ACS)

sRAGE levels in patients with ACS, defined as unstable angina, non-ST-segment elevation myocardial infarction (STEMI), and STEMI, have been presented in twelve published cohorts with diverging results (Table 1). Cai et al. and Park et al. reported elevated levels of sRAGE in patients with ACS when compared with healthy controls [43, 44]. The study by Basta et al. did not find different sRAGE levels in non-STEMI patients compared to patients with stable CAD. However, sRAGE concentrations were higher in patients with elevated cardiac Troponin I (TnI), a specific and approved biomarker of acute myocardial infarction (AMI) [45]. In the study by Raposeiras-Roubín et al., plasma samples were collected within 12 hrs after symptoms in relation to percutaneous coronary intervention (PCI) [46], whereas Cai et al. and Basta et al. collected the blood samples at a later time point [43, 45]. Raposeiras-Roubín et al. reported similar sRAGE levels in STEMI and non-STEMI patients, but increased sRAGE levels were associated with poor in-hospital prognosis [46]. The time of blood sampling was not reported. Fukushima et al. found equal sRAGE levels in patients with ACS at baseline and at 8–12 months follow-up [47]. The time of baseline sampling is not described in detail, but one could speculate that blood was drawn at randomization for different statin treatment 72 hrs after PCI.

**Figure 1:**
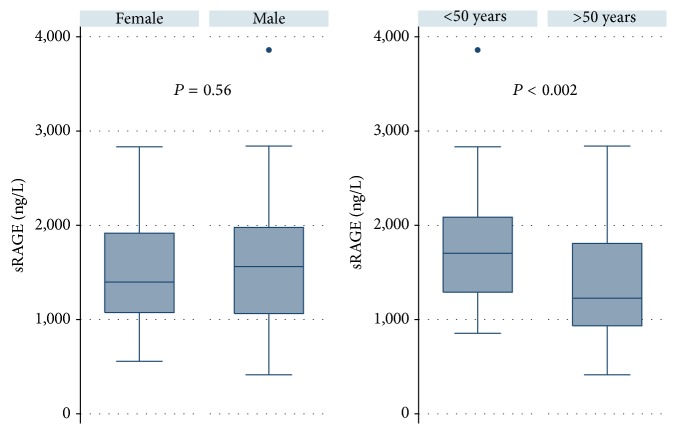
sRAGE concentrations in 100 healthy individuals. Healthy individuals are divided by gender (female (*n* = 50) and male (*n* = 50)) or by age (below 50 years (*n* = 50) and above 50 years (*n* = 50)).

In two different cohorts of STEMI patients, samples were drawn within 12 hrs after onset of symptoms and before PCI. We recently reported a fourfold increase in sRAGE levels in these STEMI patients as compared to 100 healthy individuals (described in Figure 1) [48, 49]. Successive blood samples were drawn during and after treatment of one of the STEMI cohorts, and sRAGE levels reduced almost threefold the day after PCI and decreased even further two days after PCI [49]. Interestingly, the increase of sRAGE was seen prior to TnI. The rapid decrease in sRAGE levels the day after PCI may provide valuable information in relation to diagnosis of reinfarction. Our results support the fact that sRAGE levels are elevated particularly in relation to acute ischemia, which may indicate sRAGE as an additional biomarker of AMI. In addition, the repeated measurements elucidate that sRAGE levels change over a very narrow time span in relation to acute disease. Therefore, attention to time point of sRAGE measurement is important when interpreting the results.

In contrast to the above studies, Falcone et al. found significantly decreased sRAGE levels in patients with ACS as compared to stable angina [55]. Blood samples were collected before the revascularization. Similarly, McNair et al. found lower levels of sRAGE in patients with non-STEMI compared to the controls, and time of blood sample collection was not indicated [51–54].

Only few studies have evaluated the effect of diabetes on the sRAGE levels in patients with ACS. In a group of patients (50% with type 2 diabetes (T2D)), Park et al. reported higher plasma sRAGE levels in patients with AMI than in controls, however, regardless of the presence of diabetes [44]. Similarly, Fukushima et al. reported no differences in sRAGE levels in diabetic ACS patients (30%) compared to nondiabetics with ACS [47]. Our two studies of STEMI patients included 6% and 9% diabetics and no difference in sRAGE levels was found due to diabetes [48, 49].

The ambiguity in the studies may be explained by incomparable conditions between studies; of particular importance is the time point of blood sample collection and age. A different ratio of diabetic patients within the studies described in Table 1 may also contribute to the inconclusive results as the data in diabetics with ACS are sparse.

The major source of sRAGE in relation to ACS is still not clear, but it is highly probable that it originates from the cardiomyocytes or the vascular cells in the damaged myocardium.

## 5. sRAGE in Patients with Stable CAD

Opposite to the patients with ACS, patients with stable CAD had low plasma sRAGE levels when compared to controls [35, 50, 56–58]. In a large population study including 2,571 individuals, high coronary calcium score, a risk marker of CVD, was more prevalent in the group with low sRAGE levels [59]. Furthermore, in a small prospective study of patients with suspected CAD, low sRAGE levels were predictive of future cardiovascular events after 48 months of follow-up [60]. In support, low sRAGE levels in the Atherosclerosis Risk in Communities (ARIC) Study were an indicator of future CAD after 18 years of follow-up [61]. One might speculate that the low levels of sRAGE in nondiabetic patients with stable CAD may reflect a local release of RAGE from the atherosclerotic vessels. sRAGE may capture RAGE ligands and thereby reduce measurable sRAGE in the circulation and furthermore reduce the activity in the RAGE axis.

Patients with T2D and stable CAD had significantly higher levels of sRAGE than the nondiabetic CAD patients [58, 62], and the T2D patients with high sRAGE concentrations had increased risk of CAD [63]. sRAGE levels were able to predict future CAD in T2D patients after approximately 4 years of follow-up [40]. In addition, high sRAGE levels were associated with an increased cardiovascular morbidity and mortality in T1D during follow-up [64, 65]. It may be speculated that the persistent high levels of sRAGE in diabetic patients may reflect an ongoing inflammatory and RAGE activity related to diabetes.

## 6. Measurement and Variation in sRAGE Levels

As a potential biomarker, sRAGE may be influenced by detection methods and various factors such as gender, age, and ethnicity as well as diseases and medications. In the majority of published studies, sRAGE levels have been determined with a human sRAGE enzyme-linked immunosorbent assay (ELISA) (DRG00, R&D Systems). In the enclosed datasheet, mean of sRAGE levels was 1,655 (±693 (SD)) ng/L in EDTA plasma and 1,794 (±693 (SD)) ng/L in serum in apparently healthy volunteers. An extern validation revealed a stable assay with comparable concentrations in EDTA plasma and serum samples [66]. We validated a time-resolved immunofluorometric assay (TRIFMA) using commercial human RAGE antibodies (DY1145, R&D Systems) [49] and found comparable sRAGE concentrations by TRIFMA and ELISA. We detected plasma sRAGE levels of 1,533 ng/L (±61 (SD)) in 100 healthy individuals. No difference in sRAGE levels was seen according to gender (*P* = 0.56), but age introduced a difference since individuals below the age of 50 had significantly higher levels of sRAGE than those above the age of 50 (1,759 (±86 (SD)) ng/L versus 1,378 (±78 (SD)) ng/L, *P* < 0.002, Figure 1). A negative association between age and sRAGE levels was also observed in some diabetic cohorts [42, 63] and in a population study [59].

sRAGE levels remained stable when repeatedly measured within at least 3 years in patients with and without diabetes [40, 42, 67]. A negative association between sRAGE levels and body mass index (BMI) has been found in some cohorts [42, 61] and also in one of our studies [48]. Additionally, ethnicity is reported to influence the sRAGE levels as higher sRAGE levels are reported in white compared with black individuals [40, 61, 68]. sRAGE levels may be influenced by diseases other than CAD and diabetes, for example, cancer, inflammatory diseases, neurodegenerative diseases, or chronic kidney disease [69]. Furthermore, medications may also affect sRAGE levels [70, 71].

## 7. Conclusion

Several studies indicate that RAGE activation may influence the pathogenesis and reflection in sRAGE levels in acute and stable CAD differently. The current studies demonstrated that, in nondiabetic patients, sRAGE levels are elevated in relation to ACS and sparse data indicate that diabetes does not have an additive effect in ACS patients. On the contrary, in patients with stable CAD, sRAGE levels are low in nondiabetic patients but elevated in diabetic patients which may add predictive value to recognition of future CVD.

Current data on sRAGE levels in CAD are diverging and sRAGE may be influenced by several other factors, which is why precaution must be taken with the drawn conclusions. In relation to ACS, we found the time of sampling to be of importance, which is highly relevant for evaluation as a potential biomarker. Additional mechanistic studies are needed as well as investigations of the sources and functions of sRAGE. Further clinical studies are also needed to establish the value of sRAGE as a prognostic marker in patients with ACS.

## Figures and Tables

**Table 1:** 

Patients	sRAGE	Description of main results	Reference
STEMI treated with PCI with/without remote ischemic conditioning (*n* = 191)	↑	Increased sRAGE levels with higher NYHA classification. No effect of remote ischemic conditioning on sRAGE levels and association between sRAGE and salvage index.	Jensen et al., 2015 [48]

STEMI treated with PCI (*n* = 80)	↑	Consecutive samples show high sRAGE levels prior to and immediately after PCI followed by decreased levels day 1 and day 2 after PCI. sRAGE was an independent predictor of cardiac dysfunction assessed by decreased LVEF.	Jensen et al., 2015 [49]

ACS (*n* = 208)	→	No difference in sRAGE levels at baseline and after 8–12 months after PCI. Baseline sRAGE levels were not associated with plaque progression 8–12 months after PCI.	Fukushima et al., 2013 [47]

ACS (*n* = 330), stable angina (*n* = 530)	↓	Significantly decreased sRAGE levels in ACS compared with stable angina.	Falcone et al., 2013 [55]

STEMI (*n* = 102), non-STEMI (*n* = 113)	→	No difference in sRAGE levels between STEMI and non-STEMI. Elevated sRAGE level was associated with in-hospital cardiac events.	Raposeiras-Roubín et al., 2013 [46]

Non-STEMI (*n* = 190), stable angina (*n* = 75)	→ ↑	No difference in sRAGE levels between non-STEMI and stable angina. Increased sRAGE in patients with elevated TnI.	Basta et al., 2011 [45]

ACS (*n* = 420), stable angina (*n* = 211), controls (*n* = 251)	↑	Increased sRAGE levels in ACS compared with controls.	Cai et al., 2011 [43]

AMI (*n* = 54), controls (*n* = 54)	↑	Increased sRAGE levels in patients with AMI. Diabetic patients with AMI had higher sRAGE levels than diabetic patients without AMI.	Park et al., 2011 [44]

Non-STEMI (*n* = 36), controls (*n* = 30)	↓	Lower sRAGE levels in non-STEMI compared to controls. Negative correlation between sRAGE and cTnI.	McNair et al., 2011 [51] McNair et al., 2010 [52]

Non-STEMI (*n* = 46), controls (*n* = 20)		Lower sRAGE levels in non-STEMI compared to controls. Non-STEMI with post-PCI restenosis had lower post-PCI sRAGE levels than pre-PCI levels.	McNair et al., 2010 [53]

Non-STEMI (*n* = 46), controls (*n* = 28)	↓	Lower sRAGE levels in non-STEMI compared to controls. sRAGE levels were inversely associated with the number of diseased vessels.	McNair et al., 2009 [54]

ACS: acute coronary syndrome; AMI: acute myocardial infarct; NYHA: New York Heart Association classification; LVEF: left ventricular ejection fraction; PCI: percutaneous coronary intervention; sRAGE: soluble receptor of advanced glycation end products; STEMI: ST-segment elevation myocardial infarction; TnI: Troponin I.
